# Demographic, Psychiatric, and Toxicological Factors Associated With Intentional Poisoning: A Cross‐Sectional Study of Hospital Emergency Department Registry Data

**DOI:** 10.1002/hsr2.73205

**Published:** 2026-09-09

**Authors:** Zahra Khalighi, Naser Abbasi, Elaheh Karimi, Hori Ghaneialvar, Hojjat Sayyadi, Yousef Veisani

**Affiliations:** ^1^ Department of Emergency Medicine, Associate Professor of Forensic Medicine and Clinical Toxicology School of Medicine, Shiraz University of Medical Science; ^2^ Department of Pharmacology, Faculty of Medicine, Biotechnology and Medicinal Plants Research Center Ilam University of Medical Sciences Ilam Iran; ^3^ Biotechnology and Medicinal Plants Research Center Ilam University of Medical Sciences Ilam Iran; ^4^ Non‐Communicable Diseases Research Center Ilam University of Medical Sciences Ilam Iran; ^5^ Psychosocial Injuries Research Center Ilam University of Medical Sciences Ilam Iran

**Keywords:** associated factors, attempted, emergency medicine, epidemiology, Intentional poisoning, suicide, toxicology

## Abstract

**Background and Aims:**

Intentional poisoning is a major public health challenge requiring rapid emergency intervention. This study aimed to identify demographic, psychiatric, and toxicological factors associated with intentional poisoning among patients presenting to a hospital emergency department.

**Methods:**

We conducted a retrospective cross‐sectional analysis of 658 poisoning cases recorded in a hospital emergency department poisoning registry in Ilam, Iran (March 2022–March 2023), with linked medical‐record data reviewed after the index encounter. The primary outcome was a final binary classification of intentional versus unintentional poisoning. Categorical associations were examined using Pearson chi‐square tests or Fisher exact tests as appropriate. A multivariable logistic regression model included five prespecified clinical/psychiatric variables and estimated adjusted odds ratios (aORs) with 95% confidence intervals (CIs). Analyses used Stata version 11.0 (*α* = 0.05, two‐sided).

**Results:**

Of 658 patients, 367 (55.8%) were classified as intentional poisoning and 291 (44.2%) as unintentional. Intentional poisoning was more frequent among women than men (194/279, 69.5% vs. 173/379, 45.6%; *p* < 0.001) and among urban than rural residents (295/476, 62.0% vs. 72/182, 39.6%; *p* < 0.001). In the multivariable clinical model, previous suicide attempt showed the strongest association with intentional poisoning (aOR 4.69, 95% CI 2.55–8.61, *p* < 0.001), and psychiatric disorder history remained associated (aOR 1.78, 95% CI 1.02–3.12, *p* = 0.043); current psychiatric disorder did not remain independently associated after mutual adjustment (aOR 0.91, 95% CI 0.59–1.40, *p* = 0.669). Among specific agents, high intentional‐use proportions were observed for aluminum phosphide (17/18, 94.4%), antibiotics (13/15, 86.7%), and acetaminophen (35/43, 81.4%).

**Conclusions:**

Intentional poisoning was associated with distinct demographic patterns and psychiatric vulnerability, particularly a history of previous suicide attempts. These findings support systematic mental health assessment in emergency poisoning care and prevention strategies focused on patients with established self‐harm history and access to highly lethal agents.

## 1 Introduction

Poisoning and self‐harm remain important global public health problems, contributing to preventable morbidity and mortality and to the global burden of suicide [1, 2, 3]. Intentional poisoning—the deliberate use of a toxic substance for self‐harm—represents an important mode of suicide attempt and requires timely medical and psychiatric assessment.

Intentional poisoning disproportionately affects adolescents and young adults, a period characterized by heightened psychological vulnerability [4, 5]. The convergence of psychiatric morbidity, substance experimentation, and socio‐environmental pressures during this critical window creates a complex profile that requires targeted investigation.

Globally, the toxicological profile of intentional poisoning varies considerably. In Western countries, pharmaceuticals—particularly antidepressants, benzodiazepines, and analgesics—predominate [6]. Conversely, in the Middle East, agricultural pesticides including aluminum phosphide (ALP) feature prominently due to widespread availability and high lethality [7].

Iran presents a particularly concerning picture. ALP, known locally as the “rice tablet,” remains an important agent in intentional poisoning because of its high lethality [7, 8]. Opioid‐related poisoning also remains clinically important in Iran [9].

The etiology of intentional poisoning is multifactorial. Psychiatric disorders—particularly depression and personality disorders—constitute the strongest individual‐level predictors [10]. Prior suicide attempts represent an especially potent risk factor, with meta‐analyses indicating a 30‐ to 40‐fold increased risk of completed suicide following non‐fatal self‐harm [11]. Socio‐demographic factors including unemployment, marital disruption, and urbanization have also been associated with elevated poisoning risk [12].

Despite the substantial burden of intentional poisoning in Iran, comprehensive analyses integrating demographic, psychiatric, and toxicological factors within a single emergency department population remain limited. Existing Iranian studies have often focused on individual substance classes or on demographic patterns without simultaneous consideration of psychiatric history [13, 14].

The objectives of this study were to characterize demographic differences between intentional and unintentional poisoning patients, identify psychiatric and clinical factors associated with intentional poisoning, and describe substance‐specific patterns of intentional use.

## 2 Materials and Methods

### 2.1 Study Design and Setting

This was a retrospective cross‐sectional analysis of a hospital emergency department poisoning registry with linked medical‐record data at Shahid Mostafa Khomeini Hospital, the primary tertiary referral center for poisoning cases in Ilam Province, western Iran, serving an estimated population of 630,000 residents. The source period extended from March 21, 2022, to March 20, 2023.

### 2.2 Participants and Eligibility

During the study period, 847 patients presented to the emergency department with suspected poisoning. After exclusion of records with insufficient information for analysis (*n* = 112) and patients transferred from other facilities without complete initial assessment documentation (*n* = 77), 658 patients were included in the retrospective registry/record review. Eligibility required a clinician‐confirmed poisoning diagnosis based on history, physical examination, and toxicological assessment together with inclusion in the hospital poisoning registry.

### 2.3 Diagnostic Assessment and Biological Tests

All patients underwent routine emergency evaluation including history taking, physical examination, vital‐sign measurement, electrocardiography, and laboratory/toxicological testing as clinically indicated. The final poisoning diagnosis was established by the attending emergency physician or clinical toxicologist by integrating the exposure history, clinical presentation, and available laboratory findings.

### 2.4 Determination of Poisoning Intentionality

Intentional poisoning was defined as deliberate self‐harm or suicide attempt using a poisonous substance, based on documentation by the attending physician, corroborating family history when available, or psychiatric assessment. All other final classifications were analyzed as unintentional poisoning. The final adjudicated binary analytical variable contained 367 intentional and 291 unintentional cases, and this variable was used consistently throughout the analysis.

### 2.5 Data Collection Process

The research dataset was assembled retrospectively from existing poisoning‐registry entries and linked medical records after completion of the patients' index hospital encounters. Trained research staff reviewed the records using predefined variable definitions and abstracted information on demographics, exposure circumstances, clinical and psychiatric history, and toxicological agents. When documentation was ambiguous, clarification was sought from the treating physician regarding the existing clinical record; no patients were prospectively recruited for this research review.

### 2.6 Variables and Definitions

Demographic variables included age (years), gender (male/female), residence (urban/rural), education level (illiterate, elementary, high school, diploma, academic), occupation (unemployed, freelance, government employee, student, college student, housewife, other), and marital status. For descriptive analysis, the sparse divorced/separated categories and records with unknown marital status were combined as “other/unknown,” yielding three displayed categories: single, married, and other/unknown.

Clinical history variables included: substance use history (documented history of illicit drug use or prescription medication misuse); chronic disease history (presence of any chronic medical condition including cardiovascular disease, diabetes, cancer, chronic respiratory disease, neurological disorders); psychiatric disorder history (previous diagnosis of depression, bipolar disorder, anxiety disorders, psychotic disorders, or personality disorders by a mental health professional); previous suicide attempts (documented history of one or more prior self‐harm attempts); and current psychiatric disorder (active psychiatric diagnosis at presentation confirmed by psychiatric consultation).

Toxicological variables were recorded as separate binary indicators for aluminum phosphide, tricyclic antidepressants, methadone, morphine, acetaminophen, benzodiazepines, nonsteroidal anti‐inflammatory drugs, methamphetamine/glass, agricultural pesticides, rodenticides, alcoholic beverages, tramadol, corticosteroids, antipsychotics, antibiotics, and anti‐mania medications. These categories were not mutually exclusive; a patient could have more than one recorded agent.

### 2.7 Bias Mitigation

To minimize information bias, we employed: standardized data abstraction with clearly defined variable definitions; two independent research assistants with discrepancy resolution; formal inter‐rater reliability assessment and monitoring; physician follow‐up for ambiguous cases; and all laboratory assays performed in a single accredited hospital laboratory using standardized procedures.

### 2.8 Statistical Analysis

Analyses were performed using Stata version 11.0 (StataCorp LP, College Station, TX, USA). Statistical significance was set at α = 0.05 (two‐sided). Categorical variables are presented as frequencies and percentages. Associations between demographic characteristics and poisoning intentionality were assessed with Pearson chi‐square tests. For substance‐specific analyses, each agent was treated as a separate yes/no indicator and compared with poisoning intentionality using Pearson chi‐square tests; Fisher exact tests were used when expected cell counts were < 5. Substance categories were not mutually exclusive.

For multivariable analysis, the five clinical/psychiatric characteristics shown in Table 3 (substance use history, chronic disease history, psychiatric disorder history, previous suicide attempt, and current psychiatric disorder) were entered simultaneously into a logistic regression model. Results are presented as adjusted odds ratios (aORs) with 95% CIs and Wald‐test P values. No missing values were present for the variables reported in Tables 1, 2, 3 in the supplied final analysis dataset.

**Table 1:** Demographic characteristics of patients by poisoning intentionality (*N* = 658).

Characteristic	Total N	Intentional *n* (%)	Unintentional *n* (%)	*χ* ^2^	*p* value^a^
Gender				37.18	< 0.001
Male	379	173 (45.6)	206 (54.4)		
Female	279	194 (69.5)	85 (30.5)		
Residence				26.82	< 0.001
Rural	182	72 (39.6)	110 (60.4)		
Urban	476	295 (62.0)	181 (38.0)		
Education				18.43	0.001
Illiterate	90	37 (41.1)	53 (58.9)		
Elementary	59	25 (42.4)	34 (57.6)		
High School	90	61 (67.8)	29 (32.2)		
Diploma	289	168 (58.1)	121 (41.9)		
Academic	130	76 (58.5)	54 (41.5)		
Occupation				42.74	< 0.001
Unemployed	157	83 (52.9)	74 (47.1)		
Freelance	148	60 (40.5)	88 (59.5)		
Government	10	5 (50.0)	5 (50.0)		
Student	72	54 (75.0)	18 (25.0)		
College student	26	19 (73.1)	7 (26.9)		
Housewife	168	113 (67.3)	55 (32.7)		
Other	77	33 (42.9)	44 (57.1)		
Marital Status				9.05	0.011
Single	316	195 (61.7)	121 (38.3)		
Married	226	111 (49.1)	115 (50.9)		
Other/unknown	116	61 (52.6)	55 (47.4)		

^a^ Pearson χ^2^ test (2‐sided), significance level *p* < 0.05. The “other/unknown” marital category combines divorced, separated/other, and unknown source categories.

**Table 2:** Specific toxicological agents by poisoning intentionality (*N* = 658; categories not mutually exclusive).

Substance	Exposed *N*	Intentional *n* (%)	Unintentional *n* (%)	*χ* ^2^	*p* value^a^
Aluminum phosphide (ALP)	18	17 (94.4)	1 (5.6)	11.22	< 0.001
Tricyclic antidepressant (TCA)	24	17 (70.8)	7 (29.2)	2.29	0.130
Methadone	56	27 (48.2)	29 (51.8)	1.42	0.234
Morphine	31	14 (45.2)	17 (54.8)	1.49	0.223
Acetaminophen	43	35 (81.4)	8 (18.6)	12.24	< 0.001
Benzodiazepine (BZO)	148	115 (77.7)	33 (22.3)	37.22	< 0.001
NSAIDs	38	30 (78.9)	8 (21.1)	8.78	0.003
Methamphetamine/Glass	10	3 (30.0)	7 (70.0)	—	0.117
Agricultural pesticides	25	18 (72.0)	7 (28.0)	2.77	0.096
Rodenticides	7	5 (71.4)	2 (28.6)	—	0.472
Alcoholic beverage	26	5 (19.2)	21 (80.8)	14.66	< 0.001
Tramadol (TML)	43	28 (65.1)	15 (34.9)	1.63	0.202
Corticosteroids	1	0 (0.0)	1 (100.0)	—	0.442
Antipsychotic	3	2 (66.7)	1 (33.3)	—	1.000
Antibiotics	15	13 (86.7)	2 (13.3)	5.94	0.015
Anti‐mania	8	4 (50.0)	4 (50.0)	—	0.737

^a^ Pearson χ^2^ test for 2×2 agent‐presence comparisons; Fisher exact test used when expected cell counts were < 5. Substance categories are non‐mutually exclusive.

**Table 3:** Multivariable clinical/psychiatric factors associated with intentional poisoning (*N* = 658).

Characteristic	*n* (%)	Intentional *n* (%)	Unintentional *n* (%)	Adjusted OR (95% CI)^a^	*p* value^b^
Substance use history	154 (23.4)	86 (55.8)	68 (44.2)	0.79 (0.50–1.25)	0.309
Chronic disease history	99 (15.0)	52 (52.5)	47 (47.5)	0.92 (0.56–1.53)	0.754
Psychiatric disorder history	110 (16.7)	77 (70.0)	33 (30.0)	1.78 (1.02–3.12)	0.043
Previous suicide attempt	94 (14.3)	79 (84.0)	15 (16.0)	4.69 (2.55–8.61)	< 0.001
Current psychiatric disorder	205 (31.2)	130 (63.4)	75 (36.6)	0.91 (0.59–1.40)	0.669

^a^ Adjusted odds ratios are from a single logistic regression model containing all five variables shown.

^b^ Wald test (2‐sided), significance level *p* < 0.05.

## 3 Results

A total of 658 patients with poisoning were included in the analysis. Of these, 367 (55.8%) were classified as intentional poisoning and 291 (44.2%) as unintentional. The demographic, psychiatric, and toxicological characteristics are presented below.

### 3.1 Demographic Characteristics

Significant demographic differences were observed (Table 1). Women accounted for 279 of 658 patients (42.4%); 194 of 279 women (69.5%) were classified as intentional poisoning, compared with 173 of 379 men (45.6%) (χ^2^ = 37.18, *p* < 0.001). Urban residents comprised 476 patients, with 295 (62.0%) intentional poisonings, compared with 72 of 182 rural residents (39.6%) (*χ* ^2^ = 26.82, *p* < 0.001).

Educational attainment differed by poisoning intentionality (*χ* ^2^ = 18.43, *p* = 0.001). Among the 367 intentional cases, diploma holders represented the largest group (168/367, 45.8%), followed by academically educated individuals (76/367, 20.7%), high‐school educated patients (61/367, 16.6%), illiterate patients (37/367, 10.1%), and elementary‐educated patients (25/367, 6.8%).

Occupational status also differed significantly (*χ* ^2^ = 42.74, *p* < 0.001). Housewives constituted the largest occupational group among intentional cases (113/367, 30.8%), followed by unemployed individuals (83/367, 22.6%), freelance workers (60/367, 16.3%), students (54/367, 14.7%), other occupations (33/367, 9.0%), college students (19/367, 5.2%), and government employees (5/367, 1.4%).

Marital status was associated with poisoning intentionality after the source categories were displayed as single, married, and other/unknown (*χ* ^2^ = 9.05, *p* = 0.011). Single individuals comprised 195 of 367 intentional cases (53.1%), married individuals 111 (30.2%), and the other/unknown group 61 (16.6%).

### 3.2 Toxicological Characteristics

Substance‐specific patterns are shown in Table 2. Because agent indicators were non‐mutually exclusive, percentages are calculated within each exposed group, and rows should not be summed to obtain a patient denominator. The highest proportions of intentional poisoning were observed for ALP (17/18, 94.4%), antibiotics (13/15, 86.7%), acetaminophen (35/43, 81.4%), NSAIDs (30/38, 78.9%), and benzodiazepines (115/148, 77.7%). In yes/no comparisons with the remainder of the cohort, statistically significant associations with intentionality were observed for ALP (*p* < 0.001), acetaminophen (*p* < 0.001), benzodiazepines (*p* < 0.001), NSAIDs (*p* = 0.003), antibiotics (*p* = 0.015), and alcoholic beverages (*p* < 0.001).

Alcoholic beverage exposure was predominantly associated with unintentional poisoning (21/26, 80.8%). The remaining agents did not show statistically significant yes/no associations with poisoning intentionality in the supplied dataset after applying Pearson chi‐square or Fisher exact tests as appropriate (Table 2).

### 3.3 Psychiatric and Clinical Factors

In the simultaneous five‐variable multivariable model (Table 3), previous suicide attempt showed the strongest independent association with intentional poisoning (aOR 4.69, 95% CI 2.55–8.61, *p* < 0.001). A history of psychiatric disorder also remained associated (aOR 1.78, 95% CI 1.02–3.12, *p* = 0.043).

Substance use history (aOR 0.79, 95% CI 0.50–1.25, *p* = 0.309), chronic disease history (aOR 0.92, 95% CI 0.56–1.53, *p* = 0.754), and current psychiatric disorder (aOR 0.91, 95% CI 0.59–1.40, *p* = 0.669) were not independently associated with poisoning intentionality after mutual adjustment for the five variables in the model.

## 4 Discussion

This study examined demographic, psychiatric, and toxicological factors associated with intentional poisoning among patients presenting to a hospital emergency department in western Iran. The data‐audited analysis identified previous suicide attempt as the strongest independent clinical association, with approximately 4.7‐fold higher adjusted odds of intentional poisoning. In bivariate demographic analyses, intentional poisoning was more frequent among women and urban residents; these demographic comparisons should be interpreted as descriptive associations rather than independent causal effects.

Psychiatric disorder history remained independently associated with intentional poisoning in the multivariable clinical model (aOR 1.78). In contrast, the association for current psychiatric disorder attenuated after simultaneous adjustment (aOR 0.91, *p* = 0.669), suggesting substantial overlap among current psychiatric status, psychiatric history, and prior self‐harm. The strong association with previous suicide attempt is consistent with international evidence identifying prior self‐harm as an important marker of subsequent suicidal behavior [11, 15].

Substance use history was not independently associated with intentional poisoning in the multivariable clinical model (aOR 0.79, *p* = 0.309). This finding should be interpreted in the context of local substance‐use patterns and possible under‐documentation of sensitive exposures in medical records.

ALP showed the highest observed proportion of intentional use (17/18, 94.4%) and a significant association with poisoning intentionality in the agent‐presence analysis (*p* < 0.001). Although ALP represented only 18 of 658 patients (2.7%), its high lethality and strong intentional‐use pattern support continued attention to means restriction and safe storage [16, 17].

Benzodiazepines were the most frequently recorded listed drug class (148/658, 22.5%; agent categories were non‐mutually exclusive) and had a high intentional‐use proportion (77.7%). Acetaminophen and NSAIDs also showed high intentional‐use proportions and significant yes/no associations with intentionality. These findings support safe‐storage counseling, appropriate prescribing, and public education while recognizing that the cross‐sectional design cannot establish why a particular agent was selected.

Alcoholic beverage exposure was predominantly classified as unintentional (80.8%) and was significantly associated with lower intentionality in the yes/no comparison. Methamphetamine/glass, agricultural pesticides, rodenticides, tramadol, tricyclic antidepressants, methadone, morphine, antipsychotics, corticosteroids, and anti‐mania medications did not show statistically significant associations with intentionality in the supplied dataset. Sparse categories should nevertheless be interpreted cautiously because their estimates are imprecise.

### 4.1 Strengths and Limitations

This study has several strengths, including assessment of demographic, psychiatric, and toxicological factors within a single cohort, standardized registry/record‐based data collection, and reanalysis of the final dataset to ensure consistency between reported counts and statistical estimates.

However, several limitations warrant consideration. First, the cross‐sectional design precludes causal inference; the reported associations should be interpreted as markers rather than determinants. Second, single‐center recruitment may limit generalizability to other settings. Third, reliance on registry and medical‐record documentation may lead to under‐ascertainment of sensitive variables such as psychiatric history and substance use. Fourth, toxicological agents were coded as non‐mutually‐exclusive indicators, so substance‐specific results describe associations with individual recorded agents rather than a mutually exclusive distribution of poisoning causes. Fifth, several agent categories contained very small samples, requiring cautious interpretation. Finally, poisoning intentionality was determined retrospectively from clinical documentation. Although the analysis used the final adjudicated binary classification (367 intentional and 291 unintentional), some misclassification remains possible for cases whose intent was initially ambiguous.

Future research should employ prospective, multi‐center designs with standardized data collection, including validated psychiatric assessments and detailed substance use histories. Longitudinal follow‐up would enable examination of reattempt rates and identification of modifiable factors distinguishing single‐episode from recurrent self‐harm. Qualitative studies exploring the lived experiences of individuals who attempt intentional poisoning could illuminate the social and psychological pathways underlying our quantitative findings, informing culturally tailored interventions.

## 5 Conclusion

This study shows that intentional poisoning in western Iran is associated with distinct demographic patterns and psychiatric vulnerability. Previous suicide attempt was the strongest independent clinical factor, with approximately 4.7‐fold higher adjusted odds, while psychiatric disorder history also remained associated in the multivariable clinical model. Women and urban residents had higher proportions of intentional poisoning in bivariate analyses, and several readily available or highly lethal agents showed prominent intentional‐use patterns.

These findings have practical implications for clinical care and prevention. Emergency departments should integrate systematic assessment of previous self‐harm and psychiatric history into routine poisoning care, and discharge planning should include safety counseling and coordinated mental health follow‐up when indicated. Prevention strategies should also consider access to highly lethal agents. Because this is a single‐center cross‐sectional study, these findings should be confirmed in prospective multicenter research.

## Author Contributions

**Zahra Khalighi:** validation, investigation, resources, writing – original draft, writing – review and editing. **Naser Abbasi:** conceptualization, methodology, writing – original draft, writing – review and editing, data curation. **Elaheh Karimi:** investigation, validation, writing – original draft, writing – review and editing, methodology. **Hori Ghaneialvar:** validation, investigation, writing – original draft, writing – review and editing. **Hojjat Sayyadi:** formal analysis, software, writing – review and editing, writing – original draft. **Yousef Veisani:** conceptualization, methodology, writing – original draft, writing – review and editing, supervision, project administration, visualization, funding acquisition.

## Funding

The authors have nothing to report.

## Ethics Statement

The study was conducted in accordance with the Declaration of Helsinki and was approved by the Ethics Committee of Ilam University of Medical Sciences (ethics code: IR.MEDILAM.REC.1405.017). This was a retrospective review of existing registry and medical‐record data; there was no prospective participant recruitment or study‐specific consent procedure. Use of the existing records was conducted under the approved ethics protocol.

## Consent

I confirm the corresponding author has read the journal policies and submit this manuscript in accordance with those policies.

## Conflicts of Interest

The authors declare no conflicts of interest.

## Transparency Statement

The corresponding author, Yousef Veisani, affirms that this manuscript is an honest, accurate, and transparent account of the study being reported; that no important aspects of the study have been omitted; and that any discrepancies from the study as planned have been explained.

## Data Availability

The data that support the findings of this study are available on request from the corresponding author. The data are not publicly available due to privacy or ethical restrictions. The data that support the findings of this study are available from the corresponding author upon reasonable request.
